# Predicting fascial non-closure in ventral hernia repair with transversus abdominis release: risk factors, clinical outcomes, and implications for surgical planning

**DOI:** 10.1007/s10029-025-03455-z

**Published:** 2025-08-31

**Authors:** Daphne Remulla, Kimberly P. Woo, William C. Bennett, Alvaro Carvalho, Brianna L. Slatnick, Marisa H. Blackman, Kimberly S. Miles, Clayton C. Petro, Lucas R. Beffa, Ajita S. Prabhu, Michael J. Rosen, David M. Krpata, Benjamin T. Miller

**Affiliations:** 1 Department of Surgery, Cleveland Clinic Foundation, Cleveland, OH USA; 2 Department of Biostatistics, Vanderbilt University, Nashville, TN USA; 3 Department of Surgery, Northwestern Feinberg School of Medicine, Chicago, IL USA; 4 Cleveland Clinic Lerner College of Medicine of Case Western Reserve University School of Medicine, Cleveland, OH USA

**Keywords:** Fascial closure, Ventral hernia, Transversus abdominis release, Predict, Risk, Outcomes

## Abstract

**Background:**

Posterior components separation with transversus abdominis release (TAR) reduces tension on the anterior and posterior fascial elements in complex ventral hernia repairs, but its use does not ensure complete fascial closure. This study evaluates the relationship between hernia size and anterior fascial closure success rates following TAR and identifies predictive factors for non-closure.

**Methods:**

We retrospectively analyzed 1,677 patients who underwent open ventral hernia repair with TAR and synthetic mesh placement at a single institution from 2014 to 2023. The primary outcome was the rate of overall anterior fascial closure after TAR. Secondary outcomes included the association of hernia size with fascial closure, predictors of fascial closure and wound morbidity.

**Results:**

The overall fascial closure rate was 93.9% (*n* = 1,574). Hernia width independently predicted fascial closure success, with reduced odds for widths of 15–20 cm (OR 0.39, *p* = 0.017) and > 20 cm (OR 0.05, *p* < 0.001), relative to hernias < 15 cm. History of open abdomen (OR 0.33, *p* < 0.001) and higher ASA classification (OR 0.39, *p* = 0.042) were associated with non-closure. Fascial non-closure was associated with increased wound morbidity (*p* < 0.05), while closure independently reduced odds of one-year surgical site infection (SSI) (OR 0.13; *p* < 0.001) and surgical site infections and occurrences requiring procedural intervention (SSI/O PI) (OR 0.52; *p* = 0.001).

**Conclusions:**

While excellent overall fascial closure rates were achieved among patients undergoing TAR, specific patient and hernia characteristics significantly impact success. These findings establish a reference point for closure rates by hernia width and identify high-risk populations who may benefit from preoperative adjunctive interventions.

## Introduction

For patients with large ventral hernia defects, posterior component separation with transversus abdominis release (TAR) has been a valuable technique for repair by creating a large retromuscular pocket for wide mesh overlap while facilitating the advancement of the anterior and posterior fascial elements [1]. However, despite the advantages of TAR in managing complex ventral hernias, complete anterior fascial closure remains unattainable in a subset of patients. This unsuccessful closure has been shown to increase the risk of hernia recurrence and the potential for increased postoperative complications, though the relationship between closure status and clinical outcomes remains incompletely characterized [2].

While our group has previously described outcomes in patients without fascial closure following TAR, no studies exist comparing clinical outcomes between patients with and without successful fascial closure [2]. Perhaps most importantly, predictive factors identifying patients at risk for fascial non-closure remain poorly characterized, limiting evidence-based preoperative planning and appropriate patient selection for adjunctive interventions, such as botulinum toxin injection and progressive pneumoperitoneum.

This study aims to evaluate the association between hernia size and fascial closure success rates following open ventral hernia repair with TAR, identify independent predictors of fascial non-closure and compare clinical outcomes between patients with and without successful fascial closure to guide preoperative planning.

## Methods

After receiving Institutional Review Board (IRB) approval, we conducted a retrospective analysis of prospectively collected data from the Abdominal Core Health Quality Collaborative (ACHQC) database for all adult patients who underwent an open ventral hernia repair with transversus abdominis release (TAR) and the placement of synthetic mesh in the retromuscular space at Cleveland Clinic from January 2014 to December 2023 with a minimum of one-year follow-up. The ACHQC registry is prospectively maintained with surgeon-entered data at the point of care for continuous quality improvement [3]. At our institution, all hernia surgeons routinely enter comprehensive procedural data into the ACHQC registry as part of standard clinical practice. Our institution performs ventral hernia repair with TAR without preoperative adjuncts as our standard approach to complex ventral hernia repair. By restricting our analysis to patients treated at a single institution, we examined a cohort managed with consistent surgical technique, perioperative protocols, and decision-making algorithms. This methodological approach minimizes variability that might confound outcomes assessment in multi-institutional studies.

Our institutional approach to TAR has been previously described [4]. In brief, a generous midline laparotomy is performed, followed by complete adhesiolysis and removal of previous intraperitoneal mesh, where applicable. The posterior rectus sheath is then incised approximately 0.5 cm from its medial edge and the retromuscular plane is developed superiorly to the retroxiphoid space, inferiorly to the space of Retzius, and laterally to the linea semilunaris, with careful preservation of neurovascular bundles. The posterior lamella of the internal oblique is incised just medial to the neurovascular bundles, exposing the medial aspect of the fibers of the transversus abdominis (TA) muscle. The TA muscle is then transected, and a large pre-transversalis/retromuscular plane is developed superiorly to the diaphragm, inferiorly to the myopectineal orifice and retropubic space, and laterally to the psoas muscle. Upon finishing this dissection, the previously dissected planes are joined. The posterior rectus sheaths are then reapproximated in the midline with a running 2 − 0 absorbable suture and any fenestrations in this layer are closed. A bare permanent synthetic mesh is placed in the retromuscular position, and closed suction drains are placed anterior to the mesh. The anterior fascia is re-approximated in the midline using running or figure-of-eight slowly-absorbable monofilament suture. Finally, the subcutaneous space and skin are then closed in layers with absorbable sutures.

Within the ACHQC registry, fascial closure is documented as a binary outcome, with surgeons indicating achievement of fascial closure as “yes” or “no.” In cases of fascial non-closure, the repair involves bridging the fascial defect with mesh, where the mesh spans the gap between fascial edges without primary fascial approximation. The mesh may be fixated to the fascial edges using sutures. In some cases, partial fascial closure, which in our study was classified as non-closure, may be achieved at the superior and inferior aspects of the defect, with the central portion bridged by mesh. The primary outcome was the rate of overall fascial closure after TAR defined as a complete approximation of the fascia. We elected to include all ventral hernia types (midline and parastomal) in this cohort due to the paucity of data in these patients and the ability to adjust for the presence of a stoma using appropriate statistical methods.

Secondary outcomes included the association of hernia size with fascial closure and predictors of fascial non-closure. Other outcomes of interest included comparative peri-operative outcomes between fascial closure (FC) and non-closure (FNC) groups, including 30-day serious complications, wound morbidity and reoperations at 30 days and one year. Serious complications of interest included pulmonary embolism (PE), stroke, deep vein thrombosis (DVT), sepsis, myocardial infarction (MI), acute renal failure (ARF), pneumonia, postoperative respiratory failure requiring intubation, and death. Wound morbidity was defined as surgical site infection (SSI), surgical site occurrence (SSO), surgical site infections and occurrences requiring procedural intervention (SSI/O PI). SSI was defined as a superficial, deep, or organ space infection in accordance with Centers for Disease Control (CDC) definitions [5]. SSO included any SSI, wound cellulitis, nonhealing incisional wound, fascial disruption, skin or soft tissue ischemia, skin or soft tissue necrosis, wound serous or purulent drainage, stitch abscess, seroma, hematoma, infected or exposed mesh, or development of an enterocutaneous fistula. SSOPI was defined as any SSO that required opening of the wound, wound debridement, suture excision, percutaneous drainage, or mesh removal (partial or complete) [6].

### Statistical analysis

Patient characteristics and operative details were summarized as frequencies (n) and percentages (%) for categorical variables and compared using Pearson’s Chi-squared test or Fisher’s exact test, as appropriate. Continuous variables were presented as medians with interquartile ranges (IQR, 25th–75th percentile) and compared using Wilcoxon rank sum tests.

Fascial closure rates were calculated for each centimeter increase in hernia width. To evaluate the association between hernia width and the probability of achieving fascial closure, a weighted logistic regression model was fitted with fascial closure status as the dependent variable and hernia width (in cm) as a continuous predictor. From this model, predicted fascial closure rates by hernia width were computed using the inverse-logit transformation.

To identify independent predictors of fascial non-closure, a multivariable logistic regression model and odds ratios (OR) with 95% confidence intervals (CI) were calculated, adjusting for relevant covariates such as American Society of Anesthesiologists (ASA) class, recurrent hernia status, wound class, currently active infection, presence of a stoma, history of open abdomen, history of component separation, abdominal wall SSI history and hernia size. We elected not to use hernia width as a continuous variable in this model because doing so assumes that hernia width is a linear predictor of fascial closure and would thus quantify the effect of each one cm increase in hernia size on the likelihood of fascial closure, which is not clinically accurate. Instead, we stratified hernias into three clinically relevant hernia size groups (< 15 cm, 15–20 cm, and > 20 cm) to reflect meaningful clinical distinctions in surgical complexity and expected outcomes based on our institutional experience.

Postoperative outcomes were compared between FC and NFC groups. To assess whether fascial closure status was associated with adverse wound outcomes, a multivariable logistic regression model was developed, adjusting for relevant covariates including hernia width and diabetes. Given that larger hernias may independently increase the risk of wound morbidity irrespective of fascial closure status, we included an interaction term between fascial closure and hernia width in the regression model. This allowed us to examine whether the impact of fascial closure on wound outcomes varied depending on hernia width. Statistically, the interaction term (fascial closure x hernia width) captures any non-additive relationship between these two variables, enabling a more nuanced assessment of how their combined influence impacts postoperative wound risk.

All statistical analyses were conducted using R software (version 4.0.0, Vienna, Austria), with *p* < 0.05 considered statistically significant for all comparisons.

## Results

After applying inclusion and exclusion criteria, 1,677 patients were included. Patient characteristics and operative details are summarized in Table 1. The median age was 61 years (IQR: 52–68 years) and 53.7% (*n* = 901) were female with a BMI of 32.2 kg/m^2^ (IQR: 28.2–35.9). A total of 251 patients (15.0%) had a concomitant parastomal hernia. The median hernia width was 15.0 cm (IQR: 12, 19).

**Table 1:** Patient characteristics and operative details

Variable	Overall (*N* = 1677)	Fascial Closure (*N* = 1574)	No Fascial Closure (*N* = 103)	*P*-value
Age, median (25th, 75th)	61.0 [52.0, 68.0]	61.0 [52.0, 68.0]	62.0 [54.5, 68.0]	0.43
Female Gender	901 (53.7%)	847 (53.8%)	54 (52.4%)	0.864
Race				0.209
White	1567 (93.4%)	1473 (93.6%)	94 (91.3%)	
Black or African American	69 (4.1%)	63 (4.0%)	6 (5.8%)	
Hispanic	18 (1.1%)	17 (1.1%)	1 (1.0%)	
Not Indicated	6 (0.4%)	4 (0.3%)	2 (1.9%)	
White	1567 (93.4%)	1473 (93.6%)	94 (91.3%)	
American Indian or Alaskan Native	2 (0.1%)	2 (0.1%)	0 (0%)	
Asian Native Hawaiian or Other Pacific Islander	2 (0.1%)	2 (0.1%)	0 (0%)	
Middle Eastern	10 (0.6%)	10 (0.6%)	0 (0%)	
Other or unknown	3 (0.2%)	3 (0.2%)	0 (0%)	
ASA Class				< 0.001
I	3 (0.2%)	3 (0.2%)	0 (0%)	
II	253 (15.1%)	248 (15.8%)	5 (4.9%)	
III	1366 (81.5%)	1277 (81.1%)	89 (86.4%)	
IV	55 (3.3%)	46 (2.9%)	9 (8.7%)	
BMI, median (25th, 75th)	32.2 [28.2, 35.9]	32.2 [28.4, 35.9]	32.5 [27.0, 36.1]	0.474
Nicotine Use	93 (5.5%)	88 (5.6%)	5 (4.9%)	0.925
Steroid Use	154 (9.2%)	145 (9.2%)	9 (8.7%)	1
Hypertension	1045 (62.3%)	982 (62.4%)	63 (61.2%)	0.886
Diabetes Mellitus	384 (22.9%)	362 (23.0%)	22 (21.4%)	0.793
Chronic Obstructive Pulmonary Disease	157 (9.4%)	143 (9.1%)	14 (13.6%)	0.178
History of Inflammatory Bowel Disease	164 (9.8%)	161 (10.2%)	3 (2.9%)	0.024
History of Component Separation	178 (10.6%)	154 (9.8%)	24 (23.3%)	< 0.001
History of Open Abdomen	175 (10.4%)	146 (9.3%)	29 (28.2%)	< 0.001
History of Abdominal Wall SSI	387 (23.1%)	351 (22.3%)	36 (35.0%)	0.005
Prior Prosthetic Mesh Infection	151 (9.0%)	130 (8.3%)	21 (20.4%)	0.013
Current Active Infection	26 (1.6%)	22 (1.4%)	4 (3.9%)	0.117
Recurrent hernia	950 (56.6%)	873 (55.5%)	77 (74.8%)	< 0.001
Hernia Width, median (25th, 75th)	15.0 [12.0, 19.0]	15.0 [12.0, 18.0]	23.0 [19.0, 30.0]	< 0.001
Hernia Width, mean (SD)	15.0 (6.3)	15.0 (5.6)	23.0 (9.1)	< 0.001
Hernia Length, median (25th, 75th)	23.0 [20.0 26.0]	23.0 [19.0 26.0]	28.0 [24.5, 30.0]	< 0.001
Hernia Length, mean (SD)	23.0 (5.9)	23.0 (5.7)	28.0 (6.3)	< 0.001
Wound Status				< 0.001
Clean	1254 (74.8%)	1176 (74.7%)	78 (75.7%)	
Clean-contaminated	215 (12.8%)	203 (12.9%)	12 (11.7%)	0.454
Contaminated	204 (12.2%)	192 (12.2%)	12 (11.7%)	
Dirty/Infected	4 (0.2%)	3 (0.2%)	1 (1.0%)	
Operative Time				< 0.001
0–59	2 (0.1%)	2 (0.1%)	0 (0%)	
60–119	157 (9.4%)	156 (9.9%)	1 (1.0%)	
120–179	532 (31.7%)	523 (33.2%)	9 (8.7%)	
180–239	463 (27.6%)	443 (28.1%)	20 (19.4%)	
240+	523 (31.2%)	450 (28.6%)	73 (70.9%)	
Stoma Present	265 (15.8%)	254 (16.1%)	11 (10.7%)	0.183
Fascial Closure	1574 (93.9%)	1574 (100%)	0 (0%)	< 0.001
Fascial Closure Technique				1
Absorbable Suture	1573 (93.8%)	1572 (99.9%)	1 (1.0%)	
Permanent Suture	2 (0.1%)	2 (0.1%)	0 (0%)	
Running	775 (46.2%)	775 (49.2%)	0 (0%)	
Simple Interrupted	1 (0.1%)	1 (0.1%)	0 (0%)	
Figure of Eight	856 (51.0%)	855 (54.3%)	1 (1.0%)	

BMI – Body mass index

ASA- American Society of Anesthesiologists

SSI – Surgical Site Infection

**Fig. 1:**
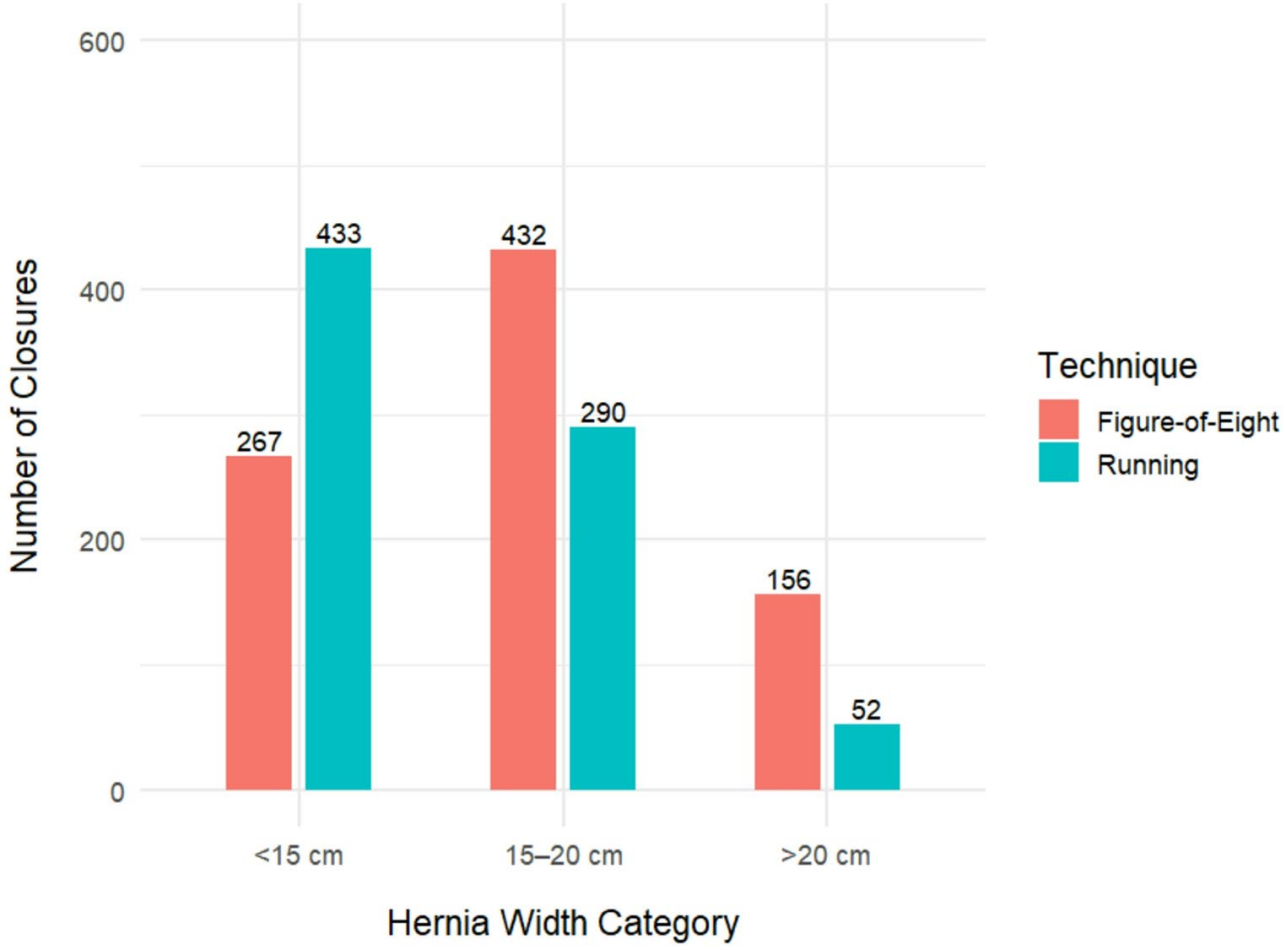
Distribution of closure technique by hernia width category. Bar heights represent the number of patients in whom either a running or figure-of-eight fascial closure technique was used, stratified by hernia width: <15 cm, 15–20 cm, and > 20 cm. Use of closure technique varied significantly by hernia width (*p* < 0.001)

Among the total cohort, fascial closure was not achieved in 103 (6.1%) patients. The NFC group had significantly larger hernias (median hernia width: 23.0 cm vs. 15.0 cm; *p* < 0.001) with a higher rate of recurrent hernias (74.8% vs. 55.5%; *p* < 0.001) compared to the FC group. NFC patients also had significantly higher rates of previous component separation (23.3% vs. 9.8%, *p* < 0.001), prior open abdomen (28.2% vs. 9.3%, *p* < 0.001), prior abdominal wall surgical site infection (35.0% vs. 22.3%, *p* = 0.005), and prior prosthetic mesh infection (20.4% vs. 8.3%, *p* = 0.013). There were no differences in patients with currently active infections or stomas present (10.7% vs. 16.1%; *p* = 0.183). Operative time was significantly longer in the NFC group, with 70.9% having operative times > 240 min compared to 28.6% in the FC group (*p* < 0.001). Mesh dimensions were correspondingly larger in the NFC group, with both mean mesh length (50.0 cm vs. 30.0 cm, *p* < 0.001) and width (50.0 cm vs. 30.0 cm, *p* < 0.001) being significantly greater than in the FC group. Nearly all complete fascial closures were done using absorbable suture (99.9%), with similar use of running (49.2%) or figure-of-eight (45.7%) suture technique [Table 1]. There was a statistically significant association between hernia width and closure technique (*p* < 0.001), with larger hernia sizes more frequently managed using the figure-of-eight closure method [Figure 1].

### Fascial closure rates and predictors of fascial Non-Closure

**Fig. 2:**
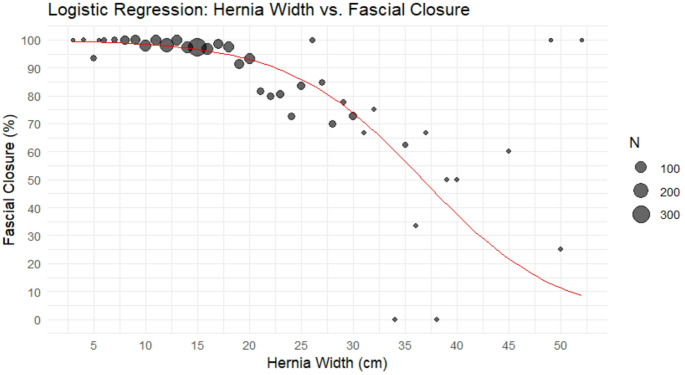
Fascial Closure Success Rates by Hernia Width. This scatter plot displays the relationship between hernia width (in centimeters) and the corresponding fascial closure rate (%). Each point represents a group of patients, with the size of the point indicating the number of cases (N) in that group. Larger circles denote greater sample sizes. The red line represents the weighted linear regression line, showing a negative association between increasing hernia width and the likelihood of fascial closure (logit(p) = 5.682 − 0.155 × Width; 95% CI: − 0.183, − 0.129, *p* < 0.00001)

**Fig. 3:**
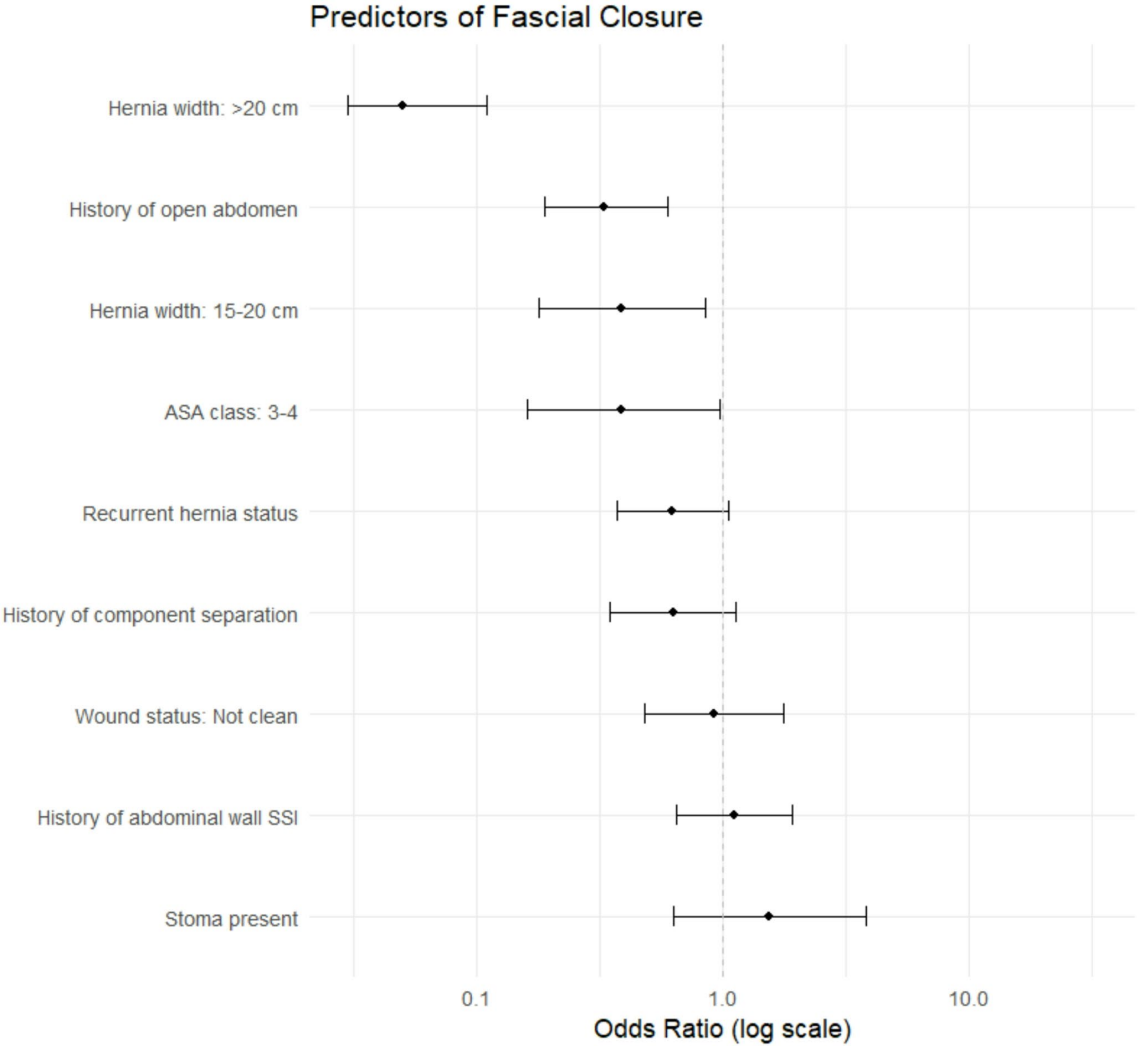
Predictors of Fascial Closure: Multivariate Regression Model. This forest plot displays the odds ratios (ORs) and 95% confidence intervals (CIs) from a multivariable logistic regression model evaluating predictors of fascial closure in patients undergoing open ventral hernia repair. Hernia size is modeled by hernia size group using the < 15 cm hernia width group as a reference. Additional covariates were history of open abdomen, ASA class, history of component separation, recurrent hernia, currently active infection, wound status, abdominal wall SSI history and presence of a stoma

Figure 2 depicts fascial closure rates by each centimeter increase in hernia width. The overall fascial closure rate was 93.9% (*n* = 1,574), with a significant inverse relationship between hernia width and rate of successful fascial closure (logit(p) = 5.682 − 0.155 × hernia width; 95% CI: − 0.183, − 0.129, *p* < 0.00001). From this model, predicted fascial closure rates are reported in Table 2. Multivariate analysis revealed that hernia size was significantly associated with fascial closure. Closure rates varied by hernia size group, with closure rates of 98.7% for hernias < 15 cm (860/869), 96.3% for hernias 15–20 cm (695/722), and 74.8% for hernias > 20 cm (199/266) (*p* < 0.001). Compared to hernias < 15 cm (reference group), hernias 15–20 cm were 61% less likely to achieve fascial closure (OR 0.39, 95% CI 0.18–0.85; *p* = 0.017), while hernias > 20 cm showed a 95% reduction in the odds of successful closure (OR 0.05, 95% CI 0.03–0.11; *p* < 0.001) [Table 3]. Independent predictors of fascial non-closure were a history of open abdomen (OR 0.33, 95% CI 0.18–0.61; *p* < 0.001) and higher ASA class (ASA class III-IV: OR 0.39, 95% CI 0.16–0.97), *p* = 0.042) [Figure 3]. In our multivariable analysis, ASA classes were grouped as I-II and III-IV due to the distribution of the cohort. Specifically, the number of patients in ASA classes I and IV was relatively small, limiting statistical power and model stability when retaining all four categories. Grouping ASA III and IV together accurately characterized these patients as higher-risk with severe systemic disease and allowed for a more clinically and statistically meaningful comparison against lower-risk patients (ASA I–II).

**Table 2:** Predicted rate of fascial closure by hernia size. This table uses the inverse-logit transformation from our weighted regression model (logit(p) = 5.682 − 0.155 × hernia width) to calculate predicted fascial closure rate

Hernia Width (cm)	Predicted Rate of Closure (%)
3	99.5
4	99.4
5	99.3
6	99.1
7	99.0
8	98.8
9	98.6
10	98.4
11	98.2
12	97.9
13	97.5
14	97.1
15	96.6
16	96.1
17	95.5
18	94.7
19	93.9
20	93.0
21	91.9
22	90.7
23	89.3
24	87.7
25	85.9
26	83.9
27	81.7
28	79.3
29	76.6
30	73.7
31	70.6
32	67.3
33	63.8
34	60.2
35	56.4
36	52.6
37	48.7
38	44.8
39	41.0
40	37.4
41	33.8
42	30.4
43	27.3
44	24.3
45	21.6
46	19.0
47	16.8
48	14.7
49	12.9
50	11.2
51	9.8
52	8.5

**Table 3:** Postoperative outcomes

Variable	Overall (*N* = 1677)	Fascial Closure (*N* = 1574)	No Fascial Closure (*N* = 103)	*P*-value
Pulmonary embolism	2 (1.9%)	21 (1.3%)	23 (1.4%)	0.988
Stroke	0 (0%)	3 (0.2%)	3 (0.2%)	1
DVT	1 (1.0%)	17 (1.1%)	18 (1.1%)	1
Sepsis	1 (1.0%)	3 (0.2%)	4 (0.2%)	0.62
Myocardial infarction	0 (0%)	6 (0.4%)	6 (0.4%)	1
Acute renal failure	2 (1.9%)	15 (1.0%)	17 (1.0%)	0.684
Pneumonia	2 (1.9%)	22 (1.4%)	24 (1.4%)	1
Respiratory failure requiring intubation	2 (1.9%)	20 (1.3%)	22 (1.3%)	0.908
Death	0 (0%)	0 (0%)	0 (0%)	1
30-day SSI	163 (9.7%)	149 (9.5%)	14 (13.6%)	0.152
Superficial	101 (6.0%)	95 (6.0%)	6 (5.8%)	0.21
Deep incisional	65 (3.9%)	55 (3.5%)	10 (9.7%)	0.025
Organ space	3 (0.2%)	3 (0.2%)	0 (0%)	1
30-day SSO	198 (11.8%)	183 (11.3%)	15 (14.6%)	0.327
Non-healing incisional wound	20 (1.2%)	15 (1.0%)	4 (4.9%)	0.008
Exposed synthetic mesh	13 (0.8%)	8 (0.5%)	5 (4.9%)	< 0.001
30-day SSOPI	184 (11.0%)	165 (10.5%)	19 (18.4%)	0.009
1-year SSI	21 (1.3%)	14 (0.9%)	7 (6.8%)	< 0.001
1-year SSO	27 (1.6%)	22 (1.4%)	5 (4.9%)	0.01
1-year SSOPI	31 (1.8%)	23 (1.5%)	8 (7.8%)	< 0.001
Reoperation at 1 year	55 (3.3%)	44 (2.8%)	11 (10.7%)	< 0.001
Major wound complication	11 (0.7%)	6 (0.4%)	5 (4.9%)	< 0.001
Recurrence	15 (0.9%)	12 (0.8%)	3 (2.9%)	0.043

DVT – Deep vein thrombosis

SSI – Surgical Site Infection

SSO – Surgical Site Occurrence

SSOPI – Surgical Site Occurrence Requiring Procedural Intervention

### Comparison of outcomes between fascial closure and non-closure

Postoperative outcomes are detailed in Table 3. There were no differences in serious complications between groups. However, the NFC group had significantly worse wound morbidity outcomes. At 30 days, the NFC group had higher rates of deep SSI (9.7% vs. 3.5%, *p* = 0.025), exposed synthetic mesh (4.9% vs. 0.5%; *p* < 0.001), and SSOPI (18.4% vs. 10.5%; *p* = 0.009). This trend persisted up to one year postoperatively, where the NFC group experienced higher rates of SSI (6.8% vs. 0.9%; *p* < 0.001), SSO (4.9% vs. 1.4%; *p* = 0.01) and SSOPI (7.8% vs. 1.5%; *p* < 0.001). Achieving fascial closure independently reduced the odds of one-year SSI (OR 0.13; *p* < 0.001) and SSI/O PI (OR 0.52; *p* = 0.001) [Table 4]. This association persisted after accounting for hernia size (interaction term, *p* = 0.769). At one year, reoperation rates were higher in the NFC group (10.7% vs. 2.8%, *p* < 0.001) with reoperations for major wound complications as the most common indication (4.9% vs. 0.4%, *p* < 0.001).

**Table 4:** Regression analysis: wound morbidity

Multivariable Logistic Regression: 1 Year SSI				
Variable	Odds Ratio	Standard Error	95% CI	P-value
Intercept	0.08	0.83	(0.01,0.38)	0.002
Fascial Closure	0.13	0.55	(0.04,0.38)	< 0.001
Hernia Width	1.02	0.03	(0.97,1.08)	0.462
**Multivariable Logistic Regression: 1 Year SSI/O PI**				
Variable	Odds Ratio	Standard Error	95% CI	P-value
Intercept	0.08	0.74	(0.02,0.35)	0.001
Fascial Closure	0.17	0.52	(0.06,0.47)	0.001
Hernia Width	1.02	0.02	(0.97,1.06)	0.459
Diabetes	2.12	0.36	(1.04,4.33)	0.039

SSI – Surgical Site Infection

SSI/O PI - Surgical Site Infection or Surgical Site Occurrence Requiring Procedural Intervention

## Discussion

In this study, we found that 93.9% of patients undergoing open ventral hernia repair with transversus abdominis release underwent successful fascial closure. Our analysis revealed a significant inverse relationship between hernia width and successful fascial closure, with a 61% reduction in odds of achieving primary fascial approximation for hernias 15–20 cm wide (OR 0.39, *p* = 0.017) and 95% reduction in odds for hernias > 20 cm wide (OR 0.05, *p* < 0.001). Multivariate analysis revealed two additional independent predictors of non-closure: history of open abdomen (OR 0.33, *p* = 0.001), and higher ASA classification (ASA class 3–4: OR 0.39, *p* = 0.042). Importantly, patients without fascial closure demonstrated significantly higher rates of short-term wound morbidity and reoperations. These findings highlight that while transversus abdominis release achieves excellent overall fascial closure rates, specific risk factors—particularly hernia width, prior open abdomen, and higher ASA classification—significantly impact fascial closure success and patient outcomes, providing valuable guidance for preoperative planning, informed consent discussions and future research.

Fascial closure after TAR has a reported rate ranging from 81 to 97.2% in the literature [7–9]. Our 93.9% overall fascial closure rate is nearly identical to that of a recent meta-analysis citing a rate of 93.8% successful fascial closure after TAR [10]. While most studies report their cohort’s mean or median hernia width and respective fascial closure rate, ours is the first to model fascial closure rates by each cm increase in hernia width [1, 11–13]. Notably, among patients achieving successful closure, larger defects were more frequently closed using figure-of-eight sutures compared to a running technique. Moreover, by modeling this relationship using a weighted logistic regression approach, we offer a means to estimate the likelihood of closure across a spectrum of defect sizes that directly correlates with differences in postoperative outcomes. While multiple factors influence surgical decision-making, the ability to objectively quantify the probability of successful fascial approximation represents a critical tool for evidence-based preoperative planning and enabling individualized risk stratification for these challenging cases.

We found that patients without fascial closure experienced markedly worse postoperative outcomes compared to patients without successful fascial closure following TAR, supporting previous findings regarding the clinical implications of failure to achieve fascial closure. Our group previously described the short-term postoperative outcomes of patients who underwent TAR without fascial closure and reported wound morbidity rates of 10% SSI, 24% SSO and 15% SSOPI at 30 days and 46% recurrence rate at a mean follow up of 20 months [2]. Our findings further expand on these results by illustrating comparatively worse wound outcomes up to one year postoperatively, as well as higher rates of reoperation compared to patients with successful fascial closure. Moreover, we identified that fascial non-closure was an independent predictor of poor wound outcomes when controlling for other variables, including hernia width. These findings highlight the significant clinical implications of failed closure on postoperative outcomes and emphasize the significance of achieving primary fascial approximation when technically feasible.

As such, several adjuncts, such as botulinum toxin injection and progressive pneumoperitoneum (PPP), have been described purporting fascial closure rates up to 100%, though high-quality studies comparing these interventions to standard approaches without adjuncts remain lacking [14, 15]. For example, in a recent systematic review analyzing 20 studies examining the efficacy of preoperative botulinum toxin and PPP, the authors found an overall 94% fascial closure rate [16]. Importantly, of the four studies with comparator arms, only two reported hernia dimensions (mean width 14.9 cm and range 8–19 cm), and none demonstrated significant differences in fascial closure rates between groups [17–20]. The risks associated with such procedures cannot be understated, particularly in cases where adjuncts may not necessarily be indicated. For example, botulinum toxin has a mandated black box warning from the US Food and Drug Administration due to the potential for systemic spread resulting in progressive muscle paralysis, weakness, and possibly death, while PPP has been associated with pneumothorax, pneumomediastinum, abdominal compartment syndrome and death [21, 22]. While these complications are uncommon, they nevertheless warrant careful consideration in the clinical decision-making process, particularly when contrasted with our study’s 96.6% rate of closure for 15 cm hernias without these adjuncts. It is therefore critically important to establish both the clinical efficacy of these interventions and robust, evidence-based criteria for patient selection that maximize benefit while minimizing risk of serious complications.

We identified three independent predictors of fascial non-closure: increased hernia width, history of open abdomen, and higher ASA classification. Hernia width is a largely intuitive risk factor for fascial non-closure as the magnitude of tissue advancement is finite, even with the use of myofascial releases. Patients with a history of open abdomen experience significant alterations to their abdominal wall, including fascial retraction, muscle atrophy, and loss of tissue elasticity, which likely impair midline approximation during repair [23]. The association between higher ASA class and fascial non-closure suggests that patient complexity plays a significant role in fascial closure status. This finding highlights an important clinical consideration that surgeons face intraoperatively: the potential trade-off between the physiological insult of elevated intra-abdominal pressure of a tight fascial closure versus the benefits of recreating the linea alba. A tight fascial closure leads to elevated intra-abdominal pressure which has been associated with increased postoperative respiratory complications [24]. Patients with higher ASA classifications may therefore be less likely to tolerate a tight fascial closure than patients with lower ASA classifications, thus surgeons may elect to leave patients such as these with a bridged repair. The physiologic changes associated with tight fascial closures and objective thresholds for opting for a bridged repair instead of complete fascial closure are ongoing areas of work for our group.

This study has several limitations. First is the inherently subjective nature of our primary outcome. Decisions regarding fascial closure rely heavily on surgeon judgment, with significant variability in what constitutes “excessive tension.” This subjectivity warrants significant consideration when interpreting our findings and underscores the need for more objective measures of fascial tension in future studies. Furthermore, our analysis is limited by the absence of other objective predictive tools and relevant variables that are not routinely captured in the ACHQC database, including hernia sac to abdominal cavity volume ratio measurements to determine loss of domain hernias, as described by Tanaka and colleagues [25]. While our analysis demonstrates associations between patient and hernia factors and fascial non-closure, the absence of these objective predictive tools represents a significant limitation, as they likely play an important role in achieving successful fascial closure [26–28]. Though fascial tension measurements and Tanaka scores may provide more precise guidance for surgical planning in this complex patient population, they still require validation in larger, prospective cohorts to confirm their predictive value and clinical utility in routine practice. Additionally, important variables that likely influence wound morbidity and postoperative complications independent of closure status were either not available in the database (such as incision length and intra-abdominal pressures) or could not be incorporated into our regression models (such as operative time). This may have resulted in unmeasured confounding, limiting our ability to definitively establish fascial closure as an independent predictor of complication risk. Though we intentionally sought to examine a cohort of patients who underwent similar preoperative decision-making and surgical techniques to reduce variability that might confound outcomes, these findings may not be generalizable to other centers. Our institution’s uniquely complex patient population, high-volume practice setting, and preference for a figure-of-eight suture technique for fascial closures likely influenced the findings presented here. Additionally, the inclusion of concomitant parastomal hernias introduces baseline differences that may not have been fully accounted for despite including stoma presence in our multivariate regression analysis. Lastly, as a retrospective analysis of a prospectively maintained database, there may be other unmeasured confounders and inconsistent reporting affecting outcomes.

Despite these limitations, our findings provide surgeons with important objective data that can provide guidance during preoperative risk assessment and patient counseling, particularly for complex cases with multiple risk factors for non-closure. Adjunct interventions such as botulinum toxin injection may have a role in carefully selected high-risk patients, though comparative studies are needed to establish whether these interventions improve closure rates beyond our high baseline success with standard techniques. Evidence demonstrating efficacy and defining appropriate patient selection criteria should guide clinical implementation. As new evidence emerges, these findings offer an important reference for future research aimed at optimizing outcomes in this challenging patient population and identifying specific patient populations in whom adjunct interventions warrant further investigation.

## Conclusion

Our findings establish an association between hernia width and fascial approximation as well as reference points for expected closure rates across different hernia sizes. The significant association between fascial non-closure and worse clinical outcomes emphasizes the critical importance of achieving midline approximation whenever possible. Additionally, we identified specific patient populations at high risk for fascial non-closure. These findings underscore the need for robust evidence examining the efficacy of preoperative adjuncts and defining clinical scenarios where such interventions may provide meaningful benefit.

## Data Availability

Data were derived from the Americas Hernia Society Quality Collaborative (ACHQC) database. Requests for access to ACHQC data is restricted and subject to approval by the ACHQC Data Use and Publications Committee.
